# Skeletal Maturity Assessment in Pediatric ACL-Reconstruction

**DOI:** 10.3390/children12091186

**Published:** 2025-09-05

**Authors:** Umile Giuseppe Longo, Mariajose Villa Corta, Federica Valente, Laura Ruzzini, Pieter D’hooghe, Kristian Samuelsson, Frank A. Cordasco, Alexander S. Nicholls

**Affiliations:** 1 Fondazione Policlinico Universitario Campus Bio-Medico, Via Alvaro del Portillo, 200, 00128 Roma, Italy; mj.villacorta@alcampus.it (M.V.C.); federica.valente@alcampus.it (F.V.); 2 Research Unit of Orthopaedic and Trauma Surgery, Department of Medicine and Surgery, Università Campus Bio-Medico di Roma, Via Alvaro del Portillo, 21, 00128 Roma, Italy; 3 Orthopedic Unit, Department of Surgery, Bambino Gesù Children’s Hospital, 00165 Roma, Italy; laura.ruzzini@opbg.net; 4 Department of Orthopaedic Surgery and Sports Medicine, Aspetar Hospital, Doha P.O. Box 29222, Qatar; pieter.dhooghe@aspetar.com; 5 Department of Orthopaedics, Sahlgrenska University Hospital, 431 30 Gothenburg, Sweden; kristian@samuelsson.cc; 6 Department of Orthopaedics, Institute of Clinical Sciences, Sahlgrenska Academy, University of Gothenburg, Göteborgsvägen 31, 405 30 Gothenburg, Sweden; 7 Sahlgrenska Sports Medicine Center, SE-411 01 Gothenburg, Sweden; 8 Sports Medicine Institute, Hospital for Special Surgery, 770 Lexington Avenue, New York, NY 10065, USA; cordascof@hss.edu; 9 Sydney Orthopaedic Research Institute, Level 2, 500 Pacific Highway, St Leonards, Sydney 2066, Australia; alexandernicholls@gmail.com; 10 The Children’s Hospital at Westmead, Hawkesbury Road, Westmead, Sydney 2145, Australia

**Keywords:** pediatric ACL injury, skeletal maturity, ACL reconstruction, growth disturbance, bone age, physeal injury, tanner staging, MRI bone age, physeal-sparing technique, pediatric orthopedics

## Abstract

**Highlights:**

**What are the main findings?**

- Skeletal maturity significantly influences surgical decision-making and outcomes in pediatric ACL reconstruction.
- A multimodal approach combining clinical, radiological, and MRI-based assessments enhances accuracy in determining skeletal maturity.

**What are the implications of the main findings?**

- Tailoring ACL reconstruction techniques to skeletal maturity levels minimizes growth disturbances and improves functional outcomes.
- An integrated assessment strategy may standardize preoperative planning, particularly for patients with ambiguous maturity status or comorbidities.

**Abstract:**

Anterior cruciate ligament (ACL) injuries in skeletally immature patients pose unique clinical and surgical challenges due to the presence of open physes and ongoing growth. In recent years, multiple surgical strategies have been developed to restore knee stability while minimizing the risk of growth disturbances. However, clinical decision-making remains complex due to the lack of consensus regarding the optimal timing, technique, and graft selection for this population. This narrative review outlines the current clinical and radiological tools used to assess skeletal maturity and explores how maturity status informs surgical approach, with particular emphasis on physeal-sparing, hybrid, and transphyseal techniques. We summarize postoperative complications—including growth disturbances and graft failure—while highlighting current guideline recommendations and ongoing controversies. Lastly, we propose a multimodal model for skeletal maturity assessment to support individualized treatment strategies and emphasize the need for standardized protocols and high-quality research to improve long-term outcomes in pediatric ACL reconstruction.

## 1. Introduction

Pediatric anterior cruciate ligament reconstruction (ACLR) presents unique challenges due to the need for accurate skeletal maturity assessment to guide surgical timing and technique selection [1,2]. Despite increasing ACL injury rates in young athletes and advances in surgical techniques, significant steps gaps exist in current maturity assessment protocols that compromise optimal decision-making and patient outcomes.

Current practice employs multiple assessment tools—including clinical methods (Tanner staging, peak height velocity) and radiological evaluations (Greulich-Pyle radiographs, MRI-based bone age)—yet discrepancies between these approaches remain common [3,4,5,6,7,8,9]. These inconsistencies contribute to inappropriate surgical timing, increased growth disturbances, and graft failures, with serious functional implications for this vulnerable population [1,2,10,11]. Moreover, the lack of standardized, evidence-based integration frameworks forces reliance on surgeon experience rather than systematic protocols.

This narrative review addresses the critical question: what are the gaps in current skeletal maturity protocols that impede optimal surgical decision-making in pediatric ACL reconstruction, and how can an evidence-based integrated framework address these clinical challenges?

We critically examine existing literature to: (1) identify specific deficiencies in current skeletal maturity assessment protocols, (2) explore how these gaps impact surgical outcomes, and (3) synthesize evidence to propose a multimodal assessment framework that integrates complementary approaches. This comprehensive analysis aims to provide clinicians with practical guidance for optimizing surgical timing decisions, reducing growth disturbances, and improving long-term functional outcomes through enhanced maturity assessment approaches.

## 2. Methods

A narrative literature review was conducted using MEDLINE, EMBASE, Scopus, CINAHL, and Google Scholar to identify relevant studies published from database inception to March 2025. Search terms included “anterior cruciate ligament”, “ACL reconstruction”, skeletal maturity”, “pediatric ACL”, “growth disturbance”, and “return to sport”. Clinical and radiological assessment methods for skeletal maturity were evaluated based on relevance to pediatric ACL surgery. Articles were selected for inclusion based on clinical applicability, originality, and contribution to the understanding of maturity assessment in surgical decision-making.

## 3. Growth Plate Structure and Assessment Complexity

Skeletal maturity, in orthopedic terminology, denotes when the growth plates (physes) of bones have fused, marking the end of longitudinal bone growth [12,13]. The growth plate (physis) is a highly organized layer of hyaline cartilage between the epiphysis and metaphysis that drives longitudinal bone growth through endochondral ossification [14,15]. This complex structure consists of four distinct zones: the resting zone (stem cell reservoir), proliferative zone (rapidly dividing cells driving elongation), hypertrophic zone (maturing and calcifying cells), and ossification zone (cartilage-to-bone conversion) [14,16]. Peripherally, the perichondral ring of Lacroix provides mechanical stability while the ossifications groove of Ranvier contributes progenitor cells for appositional growth [15].

### 3.1. Assessment Challenges Arising from Anatomical Complexity

This intricate anatomical organization creates fundamental challenges for skeletal maturity assessment. The growth plate’s multi-zonal structure undergoes continuous, dynamic changes that cannot be captured by static imaging or single-time-point evaluations [17]. The progressive transformation from cartilage to bone occurs at different rates across zones and varies significantly between individuals, making standardized assessment protocols inherently limited [17].

Furthermore, the growth plate’s cartilaginous composition renders it poorly visible on conventional radiographs, requiring indirect assessment through secondary ossification centers and metaphyseal changes [18]. This anatomical limitation forces reliance on surrogate markers rather than direct physeal evaluation, introducing assessment inaccuracies that compound clinical uncertainty.

### 3.2. Impact on Surgical Decision-Making

The growth plate’s anatomical vulnerability directly influences surgical outcomes and decision-making complexity. Any disruption to the organized zonal architecture—through drill holes, graft placement, or fixation devices—can interrupt the delicate cellular processes driving longitudinal growth [19,20]. The proliferative and hypertrophic zones are particularly susceptible to mechanical injury, with even minimal disruption potentially causing: angular deformity (abnormal varus or valgus angulation due to asymmetric growth arrest) and/or limb length discrepancy (premature partial or complete closure of the physis leads to shortening of the operated limb) [21,22,23].

This anatomical fragility explains why surgeons must carefully balance graft placement optimization with growth preservation. The inability to precisely predict individual growth plate behavior based on current assessment methods forces conservative approaches that may compromise graft positioning, or aggressive techniques that risk growth disturbance [21,22]. The anatomical complexity thus transforms every pediatric ACLR into a calculated risk-benefit decision where assessment uncertainty directly impacts surgical strategy and long-term outcomes.

## 4. Clinical Methods to Assess Skeletal Maturity

Clinical assessments remain the first step in estimating skeletal maturity for pediatric ACL reconstruction (ACLR). Their strengths lie in accessibility and speed, but their limitations—particularly in intermediate maturity ranges—create decision-making gaps that risk inappropriate surgical technique selection. Each method’s value depends on its use in the correct clinical context and, critically, in combination with complementary tools. Table 1 provides a comparative table between radiological and clinical methods.

### 4.1. Chronological Age—A Universal Reference, Never a Standalone Tool

Chronological age is universally available but does not directly reflect skeletal maturity due to wide inter-individual variation in pubertal onset [12,24,25]. Two patients of identical chronological age may differ by several Tanner stages and years of skeletal development, making sole reliance hazardous. Chronological age is most useful as an initial reference point for triaging which secondary assessments to perform. It can help identify maturity extremes—such as children under 10 years, who are almost certainly prepubertal and require physeal-sparing consideration, or older adolescents (≥16 years in boys, ≥14 years in girls), who are generally skeletally mature. However, confirmation through objective measures is mandatory in all cases.

Using chronological age alone risks performing physeal-sparing surgery unnecessarily in mature patients or premature transphyseal reconstruction in immature ones. In some community and low-resource settings, chronological age is still used in isolation, directly contributing to inappropriate technique selection. An integrated framework must eliminate this practice by requiring corroboration with at least one objective method in every case.

### 4.2. Tanner Staging (Sexual Maturity Rating)—Reliable at Maturity Extremes, Uncertain in Intermediate Stages

Tanner staging assesses pubertal development on a five-point scale based on primary and secondary sexual characteristics [26,27], with current ACLR recommendations linking stages 1–2 to physeal-sparing techniques and stage ≥ 3 to transphyseal approaches [26]. While conceptually aligned with growth-sensitive planning, real-world accuracy is inconsistent: surgeon-performed Tanner staging achieves < 60% accuracy, with the poorest performance at stage 3—the pivotal decision threshold—leading to potential misclassification in up to 40% of patients [26]. The method’s subjectivity, variability in examiner training, and its origin in a homogeneous European population [28] further limit its universal applicability.

Tanner staging can be reliable when performed by experienced pediatric clinicians in unequivocal stage 1 or stage 5 cases, where growth potential is either abundant or exhausted. However, in intermediate stages (2–4), or when assessed by non-specialists, it must be corroborated with objective measures such as bone age radiographs or MRI-based physeal evaluation to prevent inappropriate surgical technique selection. Without mandatory confirmation, up to 40% of patients risk undergoing transphyseal reconstruction too early or physeal-sparing techniques unnecessarily late, both of which can compromise outcomes. In an integrated framework, Tanner staging should serve as an initial stratification tool, triggering confirmatory imaging whenever uncertainty exists.

### 4.3. Peak Height Velocity (PHV)—Growth Momentum Indicator Without Anatomical Specificity

Peak Height Velocity (PHV) represents the period of maximal adolescent growth rate; it is used in orthopedic surgery to estimate the timing and magnitude of remaining growth potential—critical information for guiding pediatric ACL reconstruction (ACLR) decisions [29]. Identifying whether a patient is pre-, peri-, or post-PHV helps determine the appropriateness of physeal-sparing versus transphyseal surgical techniques [26].

PHV is not directly measurable in real time; instead, it is estimated retrospectively using serial height measurements plotted on standardized growth charts or predicted using anthropometric models such as the Mirwald equation, which factors sex, age, height, sitting height, and weight [30,31]. While PHV correlates with systemic skeletal maturity, its retrospective nature makes it impractical for acute injury cases. Predictive equations (Mirwald method) attempt to address this limitation but demonstrate substantial error margins and significant individual variation, particularly in athletic populations whose body proportions deviate from reference cohorts.

Moreover, PHV assesses global growth momentum but provides minimal insight into site-specific physeal maturity at the distal femur and proximal tibia—critical locations for ACL surgery [25,26]. Consequently, a patient may be classified as peri-PHV systemically but may already have partial or complete closure of knee physes, creating a significant anatomical disconnect. This anatomical disconnect limits PHV’s role as a standalone planning tool. Instead, it must be integrated with radiographic or MRI-based assessments that directly evaluate knee physeal status. This combined approach aligns global growth indicators with local skeletal maturity, addressing current protocol gaps and improving surgical decision accuracy.

### 4.4. Growth Charts and Mid-Parental Height—Contextual, Not Decisive

Growth charts plot a child’s stature against standardized percentiles to detect abnormal growth trends, while mid-parental height (MPH) estimates adult stature based on parental heights [32,33,34,35]. Both tools are useful for identifying deviations from expected growth trajectories and predicting final height, thereby providing valuable context for skeletal maturity assessment. However, neither directly measures bone age, and both are influenced by environmental, genetic, and measurement variability [30,31,32,36,37,38].

In ACLR decision-making, growth charts and MPH are most valuable as supplementary data—helping interpret outlier cases where radiographic or clinical maturity measures appear inconsistent. For example, a patient with bone age indicating immaturity but whose growth chart shows a plateau may require reassessment for possible early closure of growth plates. Despite this potential, these tools are rarely embedded in formal ACLR protocols, representing a missed opportunity to improve accuracy in borderline or atypical growth cases. Within an integrated framework, growth charts and MPH should function as modifiers, prompting re-evaluation when discrepancies arise between predicted and observed growth patterns.

### 4.5. Applicability and Limitations of Clinical Methods

Clinical methods are sufficient when:
  - Clear maturity extremes are defined by the assessment tool (e.g., obviously pre-pubertal or fully mature patients)
  - Multiple clinical indicators align consistently
  - Surgical scenarios are low-risk, where minor assessment errors have minimal consequences
  - Radiological assessment is unavailable due to resource limitations
Additional assessment is required when:
  - Clinical methods provide conflicting results (common occurrence)
  - Patients fall within intermediate maturity ranges (Tanner stages 2–4)
  - Surgical procedures are high-risk, and growth disturbances have severe consequences
  - Patient populations are atypical (e.g., athletes, ethnic minorities, endocrine disorders)
  - Surgical technique selection critically depends on precise maturity assessment

## 5. Radiological Methods to Assess Skeletal Maturity

Radiological imaging remains the cornerstone of skeletal maturity evaluation in pediatric ACLR because of its objectivity, reproducibility, and well-established growth correlations. These methods directly visualize ossification centers and physeal morphology, offering quantifiable markers for growth status. However, no single radiological approach fully captures knee-specific growth potential, and their clinical utility depends on understanding each method’s optimal use case and integration points.

### 5.1. Hand and Wrist Radiographs

Radiological methods assessing skeletal maturity commonly rely on hand-wrist imaging due to accessibility and established protocols. However, a critical limitation across these methods is their anatomical disconnect: hand-wrist maturation does not consistently reflect growth plate status at the knee—the operative site of interest for pediatric ACL reconstruction (ACLR). This mismatch reduces their precision when used in isolation for surgical decision-making and highlights the necessity of integrating complementary assessments.

#### 5.1.1. Greulich-Pyle Atlas: Widespread Use Despite Fundamental Limitations

The Greulich-Pyle (GP) atlas is among the most widely applied tools, offering rapid skeletal age estimation through comparison with standardized hand-wrist radiographs [7,39,40,41]. Its advantages include simplicity, rapid application, and broad clinical acceptance, alongside its integration into other systems, including the Tanner-Whitehouse III staging system, which demonstrates its foundational role in current practice [40,41].

Several key limitations restrict the applicability of this method for guiding ACLR decisions in pediatric populations. First, the original reference data were developed from a homogenous cohort of upper-middle-class white children in the 1930s–40s, greatly limiting relevance to today’s more diverse pediatric population [42]. Second, this radiological method over-relies on carpal bone maturation patterns that may not accurately correlate with growth in the lower extremities—the anatomical area most relevant for ACL surgery planning [40]. Third, the lack of standardized scoring protocols leading to interobserver variability, particularly problematic in borderline cases where surgical technique selection is critical [42]. Finally, the method assumes uniform skeletal maturation, disregarding well-documented variability in ossification patterns even among healthy children.

Collectively, these limitations contribute to significant uncertainty during the critical 11.5–12.5-year age range, when decisions between physeal-sparing and transphyseal surgical techniques are more consequential [43]. These factors collectively diminish the GP atlas’s standalone utility for optimizing pediatric ACL surgery timing and technique.

#### 5.1.2. Tanner-Whitehouse Method

The Tanner-Whitehouse (TW) method advances beyond the GP atlas by quantitatively scoring 20 specific hand and wrist bones to calculate a Skeletal Maturity Score (SMS), which correlates more closely with adolescent growth phases than the GP or Risser methods [29,44,45]. Its structured scoring system reduces subjectivity and improves interobserver reliability, enhancing its utility in pediatric ACLR planning. Population-specific adaptations, such as the China 05 model, further increase its clinical relevance. The TW3-RUS variant has demonstrated superior correlation with adolescent growth phases compared to both Risser staging and GP-based approaches [44].

Nevertheless, key limitations persist. Like the GP atlas, TW relies on hand-wrist radiographs and therefore also present the same anatomical disconnect problem as the other hand-wrist radiograph techniques [36,45]. Additionally, the method is time-consuming and requires specialized training, limiting routine clinical use [29,44].

Thus, while radiological methods like TW provide important maturity estimates, they may be insufficient alone—particularly in borderline cases where surgical timing is crucial [40,46]. Complementary assessments, such as clinical growth monitoring or imaging focused on the knee, are needed to guide optimal surgical decision-making in pediatric ACL reconstruction.

#### 5.1.3. Sanders Skeletal Maturity Classification

The Sanders Skeletal Maturity Classification offers a streamlined approach assessing epiphyseal fusion in the phalanges, metacarpals, and radius via hand radiographs [40,47]. Its strong predictive value for scoliosis progression, high observer reliability, and exclusion of early-maturing carpal bones enhance its clinical utility and specificity [40,48].

However, like other hand-wrist methods, Sanders does not evaluate distal femoral or proximal tibial physes, limiting its precision for ACL-specific surgical risk assessment [40]. Therefore, radiological methods like Sanders are sufficient for general maturity estimation but insufficient alone when detailed, site-specific growth assessment is essential. In such cases, supplemental tools—including clinical growth tracking and knee-focused imaging—are necessary to optimize surgical timing and technique. This highlights a key gap in current protocols: the need for integrative approaches that combine radiological data with targeted assessments to improve pediatric ACL reconstruction outcomes.

### 5.2. Elbow and Foot Radiographs

Alternative radiological sites such as the elbow and foot offer supplementary skeletal maturity information, particularly during growth phases not optimally assessed by hand-wrist imaging. However, similar to hand-wrist methods, these approaches do not directly evaluate the distal femoral and proximal tibial physes—critical anatomical regions for ACL reconstruction planning. Therefore, their findings should be integrated with knee-specific imaging and clinical assessments to provide a comprehensive maturity profile guiding surgical timing and technique selection.

#### 5.2.1. Sauvegrain Elbow Method

The Sauvegrain method assesses skeletal maturity through scoring four ossification centers visible on lateral elbow radiographs, allowing estimation of skeletal age in six-month increments [8]. This method overcomes limitations of traditional markers such as the Risser sign by providing finer temporal resolution during early puberty, a period critical for optimal ACL reconstruction timing [8,49]. Enhancements in scoring sensitivity for ages 11–15 further refine its clinical utility [8].

For pediatric ACL reconstruction, Sauvegrain offers valuable complementary maturity data that can improve surgical timing and reduce physeal injury risk [49]. However, its reliance on the elbow rather than the knee restricts its standalone applicability. Integrating Sauvegrain assessments with clinical growth indicators and knee-focused imaging remains essential to fully capture physeal maturity and tailor surgical decision-making.

#### 5.2.2. Calcaneal Assessment

Calcaneal imaging via quantitative ultrasound (QUS) and lateral radiographs presents a non-invasive, radiation-free alternative for skeletal maturity evaluation. QUS parameters such as speed of sound (SOS) and broadband ultrasound attenuation (BUA) reflect bone quality metrics related to maturation [50]. While correlations between calcaneal QUS and skeletal age demonstrate moderate reliability in females, they are notably weaker in males, contrasting with superior correlations observed in tibial QUS assessments (r = 0.76), underscoring the importance of anatomical site selection [50].

Lateral foot radiographs assessing calcaneal apophysis ossification have been structured into a six-stage scoring system, demonstrating potential for clinical use in maturity monitoring [50,51]. Despite advantages of rapidity, low cost, and accessibility, these methods face limitations including sex-dependent accuracy discrepancies, absence of standardized staging, and interpretative variability. Consequently, it currently serves best as an adjunct rather than a standalone method, particularly when traditional radiographs or MRI is unavailable or contraindicated.

### 5.3. MRI-Based Assessment

MRI provides direct, radiation-free visualization of the knee’s growth plates, epiphyseal morphology, and cartilage thickness, enabling precise assessment of physeal maturity critical for pediatric ACL reconstruction [52,53]. The Pennock Bone Age Atlas exemplifies these advances by analyzing tibial tubercle, distal femoral ossification and physeal closure, providing joint-specific maturity estimates that surpass traditional hand-wrist radiographs in precision [29,54]. This approach uncovers clinically significant discrepancies between skeletal and chronological age, particularly during the pivotal 11.5–12.5-year interval when surgical technique decisions are most sensitive [54].

Unlike traditional radiological methods that assess remote anatomical sites (e.g., hand-wrist or elbow), MRI focuses directly on the operative joint, minimizing errors caused by anatomical variability and thereby enhancing surgical planning accuracy [8,42,49]. This precision is particularly valuable in borderline or complex cases where small differences in physeal maturity influence the choice between physeal-sparing and transphyseal ACL reconstruction techniques.

Despite these advantages, the widespread use of MRI is limited by high costs, restricted availability, and longer imaging times, rendering it impractical for routine assessments. In most clinical settings, radiographic and clinical methods remain the first line of evaluation. However, integrating MRI selectively in cases where precise physeal assessment is critical can reduce the risk of growth disturbances and improve surgical outcomes.

In summary, MRI-based methods represent a substantial advancement in skeletal maturity assessment for pediatric ACLR, effectively addressing limitations of traditional approaches. When judiciously applied within a multimodal framework, MRI enhances decision-making by providing accurate, site-specific maturity data essential for optimizing surgical timing and technique.

### 5.4. Applicability and Limitations of Radiological Methods

Radiological methods are sufficient when:
  - Objective, reproducible maturity assessment is required for high-stakes orthopedic decision-making
  - Established protocols (e.g., GP atlas, TW3, Sanders, Sauvegrain) are available and clinically validated for the patient’s demographic
  - Prediction of condition progression (e.g., scoliosis) or adult height estimation requires high precision
  - Pubertal stage assessment needs accuracy within six months (e.g., spine or limb surgery timing)
  - Joint-specific growth evaluation is necessary for surgical planning (e.g., ACL reconstruction in skeletally immature patients)
  - Rapid, low-cost adjunctive imaging (e.g., calcaneal ultrasound/radiography) can enhance screening or confirm clinical impressions
Radiological methods are inadequate when:
  - The selected method is population-specific or outdated (e.g., GP atlas limitations in diverse cohorts)
  - The technique is excessively time-intensive for routine practice (e.g., TW method in busy settings)
  - The method is condition-specific and lacks broader applicability (e.g., Sanders classification in non-scoliosis cases)
  - The patient’s developmental stage falls outside the method’s optimal range (e.g., Sauvegrain method outside early puberty)
  - Imaging modality lacks standardization or has reduced accuracy in certain groups (e.g., calcaneal imaging in males)
  - High cost, limited availability, or impracticality prevent routine use (e.g., MRI in general clinical settings)

## 6. Maturity-Related Decision in Pediatric ACLR

Skeletal maturity assessment is fundamental in guiding anterior cruciate ligament reconstruction (ACLR) in pediatric patients. It directly informs surgical technique selection, timing, and graft choice, balancing the dual goals of restoring knee stability while preserving growth potential and minimizing complications.

### 6.1. Technique Selection Based on Skeletal Maturity

**Figure 1:**
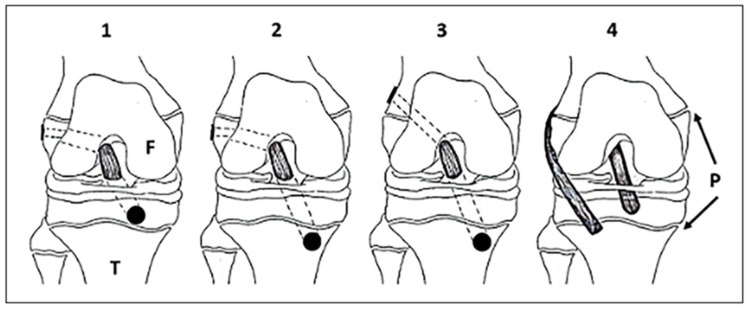
Various techniques for ACL reconstruction in the skeletally immature. Physeal sparing (1), partial transphyseal (2), transphyseal (3), and extraphyseal (4). Dotted lines represent the tunnel path. Reproduced with permission from Vavken and Murray [55].

Selecting the appropriate ACLR technique hinges on the patient’s remaining growth and skeletal age. The choice must reconcile ligament stability with growth preservation, guided by accurate maturity assessment. Figure 1 provides a visual representation of the following techniques.

**Physeal-sparing techniques (Early Skeletal Immaturity, Tanner 1–2):**

Ideal for patients with significant growth remaining, physeal-sparing methods confine graft placement to the epiphyses or use extra-articular grafts, avoiding injury to open physes. This minimizes risks of limb-length discrepancies and angular deformities [1,17,56,57]. Although technically demanding and reliant on advanced imaging, these techniques are the safest option for immature knees. Limitations include a slightly higher graft failure rate and limited long-term durability data, necessitating careful patient selection and follow-up [17,58]. This approach directly addresses the challenge of balancing ligament stability with preservation of growth potential in pediatric ACL reconstruction.

**Hybrid and Partial Transphyseal techniques for Intermediate Maturity**

Combining physeal-sparing and transphyseal elements, hybrid techniques drill through the tibial physis while sparing the femoral physis—key for longitudinal growth—allowing more anatomic graft placement [17,52]. They better replicate native ACL biomechanics and simplify tibial fixation compared to fully epiphyseal approaches, particularly in patients with narrow femoral epiphyses [17,57]. Although residual risk of growth disturbance persists, especially with substantial growth remaining, careful maturity evaluation and tunnel orientation optimize outcomes [44,59,60,61,62]. This pragmatic strategy balances anatomical restoration with growth preservation, central to maturity-informed surgical planning.

**Transphyseal techniques for Near Skeletal Maturity**

Indicated for adolescents nearing skeletal maturity (males ~15–16 years, females ~13–14 years), transphyseal reconstruction drills tunnels through both femoral and tibial physes, enabling anatomic graft placement mirroring adult techniques [17,57]. By drilling through both femoral and tibial physes, it enables anatomic graft placement that mirrors adult techniques, enhancing knee stability and function. To reduce residual physeal injury risk, modern protocols use soft-tissue grafts, vertical tunnel orientation, and fixation methods that avoid compressing growth plates [17,52,63]. Although historically linked to growth complications, current evidence supports its safety and effectiveness with careful surgical planning [57,64,65]. Transphyseal ACLR thus represents the optimal approach for mature patients, underscoring precise maturity assessment as the cornerstone of surgical decision-making.

### 6.2. Timing Considerations: When to Delay vs. Proceed

Skeletal maturity critically shapes timing decisions in pediatric ACLR by balancing two competing risks: early surgery risks physeal injury, while delayed surgery increases secondary joint damage from instability. Delaying reconstruction until near skeletal maturity reduces growth plate disruption but leaves the joint vulnerable to meniscal tears and cartilage damage, which compromise long-term function [10,66,67]. Conversely, early intervention stabilizes the knee and prevents secondary injuries but increases growth disturbance risk if performed without precise maturity evaluation and appropriate technique selection [65,68].

Accurate, multimodal maturity assessment—combining clinical staging (e.g., Tanner), radiographs, and MRI—enables individualized timing by quantifying growth remaining and physeal status. This allows surgeons to identify the optimal surgical window that minimizes growth-related complications while preventing joint deterioration.

Shared decision-making with patients and families, grounded in this maturity-based risk assessment, ensures treatment aligns with clinical and psychosocial factors, maximizing functional outcomes.

### 6.3. Graft Selection and Maturity

Graft choice in pediatric ACLR must be tailored to skeletal maturity to preserve growth plate integrity and optimize long-term outcomes.

Soft-tissue autografts (hamstring, quadriceps tendons) are preferred in immature patients as they avoid physeal injury linked to bone–tendon–bone grafts, especially when fixation crosses open physes [15,17,52]. Physeal-sparing techniques use grafts routed without transphyseal drilling, while transphyseal methods require soft-tissue grafts combined with careful tunnel placement [17,52]

Excessive graft tension can compress the physis, causing a ‘tenoepiphysiodesis’ effect that slows bone growth and risks deformity [15]. Fixation hardware crossing open physes—particularly interference screws in the tibia—has been associated with physeal bone bridge formation, tethering growth and causing angular or length discrepancies [15,17]. Filling tunnels completely with soft tissue graft reduces this risk compared to empty tunnels [17].

Notably, quadriceps tendon grafts in the tibia show higher association with physeal bone bridges, highlighting graft selection’s critical role for patients with substantial growth remaining [15].

Therefore, graft choice, tensioning, and fixation methods must be carefully matched to the patient’s skeletal maturity. This tailored approach minimizes growth disturbances while maintaining graft function, directly impacting surgical planning and long-term outcomes in pediatric ACL reconstruction.

## 7. Post-Operative Complications in Pediatric ACLR

Pediatric ACL reconstruction presents unique challenges due to open growth plates and varying skeletal maturity. Complications such as growth disturbances, graft failure, and secondary meniscal injuries are closely influenced by how accurately maturity is assessed and integrated into surgical planning.

### 7.1. Growth Disturbances

Growth-related issues—including limb-length discrepancies and angular deformities—occur more frequently in patients with substantial growth remaining. Studies report a 66.7% incidence of disturbances in those with over five years of growth left, compared to minimal risk in patients nearing skeletal maturity [35]

The risk of growth disturbance varies by technique: one review observed a higher incidence after physeal-sparing procedures (5.8%) compared to transphyseal reconstructions (1.9%) [35]. Conversely, limb overgrowth > 20 mm has also been associated with all-epiphyseal techniques [69]. This variation highlights the necessity of precise maturity assessment to inform technique choice and minimize growth plate injury.

### 7.2. Graft Failure and Meniscal Injuries

Graft failure rates in pediatric patients are comparable to adults but are influenced by factors linked to skeletal maturity, such as premature return to sport and activity intensity [35]. Delaying surgery to avoid growth risks can increase secondary meniscal injuries, seen in up to 22% of conservatively managed cases [70]. Accurate maturity evaluation is therefore essential to balance timing and technique, reducing periods of instability that predispose to further injury.

Complication patterns demonstrate the integral role of skeletal maturity assessment in pediatric ACLR. By guiding individualized timing and surgical strategy, precise maturity evaluation directly contributes to reducing growth disturbances and improving functional outcomes, addressing the core challenges highlighted by the research question.

## 8. Current Guidelines and Expert Consensus

Expert societies like ESSKA-ISAKOS prioritize restoring knee function, preventing secondary injuries, and minimizing growth plate damage in pediatric ACL reconstruction. Across techniques, they recommend exclusively using soft-tissue autografts and avoiding hardware or bone plugs crossing open physes. Tunnel placement should minimize physeal injury via vertical or central trajectories. Surgical indications include persistent instability after rehab and repairable meniscal or osteochondral lesions [71].

The PRiSM Society highlights significant practice variability, especially in adolescents with moderate growth remaining (SMR stage 3), where optimal technique and timing are less defined. Although growth disturbances are uncommon, ongoing reports stress the need for cautious surgical planning and refinement [72,73].

AOSSM underscores the critical role of precise skeletal maturity assessment—using bone age, MRI, and alignment radiographs—to guide technique choice and monitor complications. Current pediatric ACL algorithms favor physeal-sparing methods in early maturity stages (SMR 1–2) and transphyseal techniques near skeletal maturity (SMR 4), reflecting a balance between growth preservation and biomechanical reliability [21,22].

However, consensus on the best assessment methods remains limited, and decision-making in transitional cases (SMR 3) is inconsistent due to scarce high-quality prospective data. These gaps highlight the urgent need for validated, maturity-specific algorithms—like the integrated model proposed here—to improve surgical precision and patient outcomes [73,74].

## 9. Proposed Integration Model for Skeletal Maturity Assessment in Pediatric ACLR

Discrepancies between clinical and radiological maturity assessments are common; for instance, a patient may appear clinically mature yet still demonstrate significant skeletal growth on imaging. To address this challenge, we propose a multimodal assessment approach that integrates clinical and radiological data to guide individualized surgical planning and optimize outcomes.

### 9.1. Assessment Algorithm

All pediatric ACL patients should undergo two initial evaluations: a clinical assessment and a radiological assessment.
  - The clinical assessment includes a detailed history and physical examination to determine chronological age and Tanner stage. When possible, previous records should be reviewed to estimate peak height velocity.
  - The radiological evaluation consists of knee radiographs (anteroposterior and lateral views) to assess physeal status. Closed physes typically allow proceeding directly to transphyseal reconstruction, as outcomes in this subgroup align with adult results.
For patients with open physes, the assessment becomes more nuanced:
  - **Early Maturity (Tanner stages I–II):** Bone age should be assessed via Greulich-Pyle hand and wrist radiographs or elbow ossification. When skeletal and chronological ages differ by less than one year, surgical planning can favor physeal-sparing techniques, minimizing growth disturbance risk.
  - **Intermediate Maturity (Tanner stage III):** Patients with some remaining growth should have bone age assessment combined with clinical markers such as secondary sexual characteristics and peak height velocity. Concordant findings support proceeding with hybrid or partial-transphyseal techniques, balancing anatomical reconstruction with growth preservation.
  - **Discordant or Complex Cases:** When clinical and radiological assessments disagree by more than one year or stage, or when there is a history of growth disorders, additional specialized evaluations are warranted before surgery. These may include MRI-based bone age assessment, endocrinology consultation (e.g., growth hormone and IGF-1 evaluation), or genetic testing if indicated. These advanced methods are reserved for cases where the standard algorithm is insufficient, given their higher cost and complexity.

### 9.2. Risk Stratification Based on Assessment Results

Based on the integration of assessment data, patients are stratified into three risk categories to guide surgical planning and monitoring:

- **Low Risk:** Patients with consistent assessment findings or discrepancies within one year/stage, no history of growth disturbances, and clear physeal status. These patients proceed with surgical techniques appropriate to their maturity level, followed by routine postoperative follow-up.
- **Moderate Risk:** Patients with discrepancies of 1–2 years/stages or borderline maturity (e.g., Tanner stage III) are best managed with conservative hybrid or partial transphyseal techniques. They require enhanced postoperative monitoring due to a moderate risk of growth disturbances.
- **High Risk:** Patients with major discrepancies between assessments, a history of growth disorders, or unclear physeal imaging findings require shared decision-making involving the patient and family. Surgical options should prioritize growth preservation, typically favoring physeal-sparing approaches, and postoperative monitoring must be intensive.

### 9.3. Quality Assurance Protocol

All assessment data, including specific values and any inter-method discrepancies, should be meticulously documented in the patient’s clinical record. The assigned risk category and the rationale for surgical timing and technique must be clearly recorded, along with a tailored postoperative monitoring plan.

To ensure reliability, all patients undergo at least one basic clinical and one radiological evaluation. Borderline or high-risk cases benefit from dual assessments and senior clinician review to optimize decision-making.

### 9.4. Validation and Continuous Improvement

Implementation of this multimodal framework must be accompanied by systematic data collection to validate the algorithm. Key metrics include the accuracy of maturity assessment relative to growth outcomes, surgical decision impact, complication rates (particularly growth disturbances and graft failures), and cost-effectiveness.

Ongoing research should refine the model based on outcome data, reliability studies, and user feedback, ensuring continual updates to assessment methods and training protocols. This evidence-based framework aims to standardize skeletal maturity assessment while maintaining flexibility for diverse clinical settings, ultimately improving surgical decision-making and patient outcomes in pediatric ACL reconstruction.

## 10. Limitations and Controversies

Despite advances in pediatric ACL reconstruction, significant challenges remain, primarily surrounding the accurate assessment of skeletal maturity. Currently, no universally accepted gold standard exists, resulting in wide variability in assessment accuracy, reliability, and clinical applicability across institutions and studies [75,76]. Clinical methods are influenced by individual variation in maturation rates, while radiological approaches—though more objective—pose ethical concerns due to radiation exposure, especially in younger children [77]. This variability complicates surgical planning and risk stratification, essential for balancing growth preservation with optimal joint stability.

The timing of surgical intervention remains particularly contentious. Early reconstruction can reduce secondary meniscal and chondral injuries and improve knee stability, but if performed prematurely, it risks growth plate damage. Conversely, delaying surgery to confirm skeletal maturity increases the risk of persistent instability and re-injury [66,78]. This delicate balance is further challenged by variability in surgical expertise, technique selection, and institutional protocols, leading to heterogeneous outcomes that limit the generalizability of findings [10,44].

Methodological limitations also restrict current knowledge. Many studies suffer from heterogeneous designs, small sample sizes, inconsistent maturity staging, and lack of long-term prospective data. Additionally, narrative reviews without systematic methodologies introduce potential bias and reduce reproducibility. Rapidly evolving surgical and imaging technologies may not yet be adequately represented in the literature, further complicating evidence synthesis.

Collectively, these issues underscore the urgent need for standardized, validated protocols for skeletal maturity assessment and prospective, multicenter research using unified outcome measures. Such efforts are critical to refining clinical decision-making, optimizing timing and technique selection, and ultimately improving long-term functional and growth-related outcomes in pediatric ACL reconstruction.

## 11. Future Directions

Integrating clinical and radiological assessments—such as MRI-based physeal mapping, the Greulich-Pyle atlas, and emerging AI-powered bone age evaluation systems—can enhance diagnostic precision, reduce subjectivity, and limit radiation exposure in skeletal maturity evaluation [78,79]. Excluding skeletally mature patients from pediatric ACLR outcome analyses is essential to accurately assess growth-related complications.

Heightened surgeon awareness of growth disturbance risks should drive the adoption of standardized post-operative surveillance protocols. Longitudinal monitoring across larger cohorts will enable earlier detection of subclinical and clinically significant growth disturbances, deepening understanding of surgical impact on immature skeletons.

Future research must prioritize prospective, multicenter trials validating multimodal assessment algorithms and comparing techniques across maturity stages. These studies should include inter-observer reliability testing and cost-effectiveness analyses tailored to risk stratification groups. Additionally, long-term outcome evaluations correlating assessment accuracy with growth disturbances, graft failure, and re-injury rates are needed.

Personalized surgical planning incorporating advanced 3D imaging and growth modeling, alongside rehabilitation and return-to-sport protocols aligned with skeletal maturity, could reduce re-injury and improve functional recovery [80,81]. Finally, collaborative multicenter registries with standardized data collection and reporting are critical to generate robust, high-quality evidence, guiding optimized, evidence-based clinical decision-making in pediatric ACL reconstruction [82,83].

## 12. Conclusions

The variability and limitations inherent in current skeletal maturity assessment methods directly contribute to suboptimal surgical timing, increased growth disturbances, and higher graft failure rates in pediatric ACL reconstruction. Our analysis demonstrates that reliance on single-method evaluations—whether clinical or radiological—can lead to significant misclassification of skeletal maturity, exposing patients to either premature intervention risks or delays that increase secondary joint damage.

Implementing a standardized, multimodal maturity assessment framework that combines clinical staging, radiographic evaluation, and advanced imaging when indicated offers a more reliable basis for personalized surgical planning. This integrated approach enables accurate risk stratification, guiding technique selection tailored to the patient’s true developmental status. Such precision mitigates the trade-off between restoring knee stability and preserving growth plate integrity, addressing the core challenge in pediatric ACLR.

Moreover, the proposed framework’s risk-based stratification informs not only surgical timing but also postoperative monitoring and rehabilitation, reducing the incidence of growth-related complications and improving long-term functional outcomes. However, the success of this model depends on prospective validation through multicenter studies and the incorporation of emerging technologies like AI-driven imaging analysis to further refine maturity assessment.

In conclusion, advancing pediatric ACL reconstruction outcomes hinges on transitioning from fragmented, experience-based decision-making to evidence-based, integrated protocols for skeletal maturity assessment. This paradigm shift promises to minimize preventable complications, optimize surgical interventions, and ultimately enhance quality of life for skeletally immature patients with ACL injuries.

## Figures and Tables

**Table 1:** Summary of commonly used clinical and radiological methods for skeletal maturity assessment in pediatric patients.

Method	Modality	Age Range	Optimal Clinical Application	Clinical Scenarios Requiring Combined Modalities	Advantages	Limitations
**Chronological Age**	Clinical (birth date)	All pediatric ages	Initial screening; identification of maturity extremes (<10 years or >16/14 years)	Sole reliance for surgical timing or borderline cases	Universally available; no associated cost; readily accessible	Poor correlation with skeletal maturity; high inter-individual variability
**Tanner Staging**	Clinical examination	Puberty (approx. 8–16 years)	Clear Tanner stage 1 or 5; assessment by trained pediatric specialists	Intermediate stages (2–4); non-specialist examiners; cultural variability	Non-invasive; directly linked to pubertal development	Subjective assessment; moderate accuracy; potential for misclassification
**Peak Height** **Velocity (PHV)**	Anthropometric measurement and growth charts	Puberty (approx. 9–16 years)	Longitudinal growth monitoring; elective surgical planning	Acute injuries; athletic populations; requirement for site-specific maturity assessment	Reflects biologically relevant growth timing	Retrospective or model-based estimation; lacks anatomical specificity
**Growth Charts and Mid-Parental Height**	Clinical records and parental data	All pediatric ages	Monitoring abnormal growth patterns; supplementary context for maturity assessment	Direct use for surgical decision-making; atypical growth patterns requiring verification	Provides longitudinal growth context; easily obtainable	Indirect skeletal maturity indicator; influenced by genetic and environmental factors
**Greulich–Pyle** **Atlas**	Radiograph (left hand-wrist)	Childhood to adolescence (approx. 2–18 years)	Rapid, low-resource maturity estimation	Need for precise growth phase determination for surgical timing	Widely accessible; rapid application; minimal training required	Subjective interpretation; outdated reference population; limited precision
**Tanner–Whitehouse (TW3–RUS)**	Radiograph (left hand-wrist)	Childhood to adolescence (approx. 2–18 years)	Detailed growth phase prediction, especially early to mid-puberty cases	Need for knee-specific physeal status confirmation	Quantitative and reproducible scoring; adaptable to populations	Time-intensive; requires specialized training; limited to hand–wrist assessment
**Sanders Staging**	Radiograph (left hand-wrist)	Puberty (approx. 8–16 years)	Efficient peak height velocity estimation in high-volume clinical settings	Necessity for anatomical site-specific validation	Streamlined evaluation; strong correlation with growth phases	Indirect skeletal maturity assessment; potential staging overlap
**MRI-Based Knee Assessment**	MRI (distal femur and proximal tibia)	Childhood to adolescence (approx. 10–16 years)	Assessment of local growth plate status in borderline or complex cases	Discordance between clinical and hand–wrist findings	Direct visualization of relevant physes; no radiation exposure	High cost; limited availability; requires expert interpretation
**Knee** **Radiographs**	Radiograph (knee)	Childhood to adolescence (approx. 10–16 years)	Site-specific skeletal maturity assessment when MRI is unavailable	Requirement for corroboration of systemic growth trends	Cost-effective; accessible imaging modality	Exposure to ionizing radiation; limited soft-tissue detail

## Data Availability

No new data were created or analyzed in this study. Data sharing is not applicable to this article.

## Abbreviations

The following abbreviations are used in this manuscript:

ACL	Anterior Cruciate Ligament
ACLR	Anterior Cruciate Ligament Reconstruction
AI	Artificial Intelligence
ITB	Iliotibial band
MRI	Magnetic Resonance Imaging
SMR	Sexual Maturity Rating
TW3	Tanner-Whitehouse 3 Method
U.S.	United States
UK	United Kingdom

## References

[1] Kocher M.S., Garg S., Micheli L.J. (2005). Physeal sparing reconstruction of the anterior cruciate ligament in skeletally immature prepubescent children and adolescents. J. Bone Jt. Surg. Am..

[2] Dunn K.L., Lam K.C., Valovich McLeod T.C. (2016). Early Operative Versus Delayed or Nonoperative Treatment of Anterior Cruciate Ligament Injuries in Pediatric Patients. J. Athl. Train..

[3] De Sanctis V., Elhakim I.Z., Soliman A.T., Elsedfy H., Elalaily R., Millimaggi G. (2016). Methods for Rating Sexual Development in Girls. Pediatr. Endocrinol. Rev..

[4] Tinggaard J., Mieritz M.G., Sørensen K., Mouritsen A., Hagen C.P., Aksglaede L., Wohlfahrt-Veje C., Juul A. (2012). The physiology and timing of male puberty. Curr. Opin. Endocrinol. Diabetes Obes..

[5] Cole T.J. (2020). Estimating peak height velocity in individuals. Ann. Hum. Biol..

[6] Khadilkar V., Phanse S. (2012). Growth charts from controversy to consensus. Indian J. Endocrinol. Metab..

[7] Pyle S.I., Waterhouse A.M., Greulich W.W. (1971). Attributes of the radiographic standard of reference for the National Health Examination Survey. Am. J. Phys. Anthropol..

[8] Diméglio A., Charles Y.P., Daures J.P., de Rosa V., Kaboré B. (2005). Accuracy of the Sauvegrain method in determining skeletal age during puberty. J. Bone Jt. Surg. Am..

[9] Neal K.M., Shirley E.D., Kiebzak G.M. (2018). Maturity Indicators and Adolescent Idiopathic Scoliosis: Evaluation of the Sanders Maturity Scale. Spine.

[10] Fabricant P.D., Jones K.J., Delos D., Cordasco F.A., Marx R.G., Pearle A.D., Warren R.F., Green D.W. (2013). Reconstruction of the anterior cruciate ligament in the skeletally immature athlete: A review of current concepts: AAOS exhibit selection. J. Bone Jt. Surg. Am..

[11] Mizuta H., Kubota K., Shiraishi M., Otsuka Y., Nagamoto N., Takagi K. (1995). The conservative treatment of complete tears of the anterior cruciate ligament in skeletally immature patients. J. Bone Jt. Surg. Br..

[12] Malina R.M., Eisenmann J.C., Cumming S.P., Ribeiro B., Aroso J. (2004). Maturity-associated variation in the growth and functional capacities of youth football (soccer) players 13–15 years. Eur. J. Appl. Physiol..

[13] Yuan J.T., Furdock R.J., Benedick A., Liu R.W. (2022). Estimating Skeletal Maturity by Segmented Linear Modeling of Key AP Knee Radiographic Parameters. J. Pediatr. Orthop..

[14] Lazar L., Phillip M. (2012). Pubertal disorders and bone maturation. Endocrinol. Metab. Clin. N. Am..

[15] Seil R., Weitz F.K., Pape D. (2015). Surgical-experimental principles of anterior cruciate ligament (ACL) reconstruction with open growth plates. J. Exp. Orthop..

[16] Katsimbri P. (2017). The biology of normal bone remodelling. Eur. J. Cancer Care.

[17] Mallinos A., Jones K. (2024). The Double-Edged Sword: Anterior Cruciate Ligament Reconstructions on Adolescent Patients-Growth Plate Surgical Challenges and Future Considerations. J. Clin. Med..

[18] Li X., Shi S., Chen J., Zhong G., Liu Z. (2018). Leptin differentially regulates endochondral ossification in tibial and vertebral epiphyseal plates. Cell Biol. Int..

[19] Kocher M.S., Smith J.T., Zoric B.J., Lee B., Micheli L.J. (2007). Transphyseal anterior cruciate ligament reconstruction in skeletally immature pubescent adolescents. J. Bone Jt. Surg. Am..

[20] Lawrence J.T., Argawal N., Ganley T.J. (2011). Degeneration of the knee joint in skeletally immature patients with a diagnosis of an anterior cruciate ligament tear: Is there harm in delay of treatment?. Am. J. Sports Med..

[21] Frank J.S., Gambacorta P.L. (2013). Anterior cruciate ligament injuries in the skeletally immature athlete: Diagnosis and management. J. Am. Acad. Orthop. Surg..

[22] Falciglia F., Panni A.S., Giordano M., Aulisa A.G., Guzzanti V. (2016). Anterior cruciate ligament reconstruction in adolescents (Tanner stages 2 and 3). Knee Surg. Sports Traumatol. Arthrosc..

[23] Koman J.D., Sanders J.O. (1999). Valgus deformity after reconstruction of the anterior cruciate ligament in a skeletally immature patient. A case report. J. Bone Jt. Surg. Am..

[24] Tanner J.M. (1983). Assessment of Skeletal Maturity and Prediction of Adult Height (TW2 Method).

[25] Marshall W.A., Tanner J.M. (1969). Variations in pattern of pubertal changes in girls. Arch. Dis. Child..

[26] Slough J.M., Hennrikus W., Chang Y. (2013). Reliability of Tanner staging performed by orthopedic sports medicine surgeons. Med. Sci. Sports Exerc..

[27] Koopman-Verhoeff M.E., Gredvig-Ardito C., Barker D.H., Saletin J.M., Carskadon M.A. (2020). Classifying Pubertal Development Using Child and Parent Report: Comparing the Pubertal Development Scales to Tanner Staging. J. Adolesc. Health.

[28] Espeland M.A., Gallagher D., Tell G.S., Davison L.L., Platt O.S. (1990). Reliability of Tanner stage assessments in a multi-center study. Am. J. Hum. Biol..

[29] Okuda A., Shigematsu H., Fujii H., Iwata E., Tanaka M., Morimoto Y., Masuda K., Yamamoto Y., Tanaka Y. (2020). Reliability Comparison between “Distal Radius and Ulna” and “Simplified Tanner-Whitehouse III” Assessments for Patients with Adolescent Idiopathic Scoliosis. Asian Spine J..

[30] Mirwald R.L., Baxter-Jones A.D., Bailey D.A., Beunen G.P. (2002). An assessment of maturity from anthropometric measurements. Med. Sci. Sports Exerc..

[31] Baxter-Jones A.D. (1995). Growth and development of young athletes. Should competition levels be age related?. Sports Med..

[32] de Onis M., Onyango A.W., Borghi E., Siyam A., Nishida C., Siekmann J. (2007). Development of a WHO growth reference for school-aged children and adolescents. Bull. World Health Organ..

[33] Grimberg A., DiVall S.A., Polychronakos C., Allen D.B., Cohen L.E., Quintos J.B., Rossi W.C., Feudtner C., Murad M.H., Drug and Therapeutics Committee and Ethics Committee of the Pediatric Endocrine Society (2016). Guidelines for Growth Hormone and Insulin-Like Growth Factor-I Treatment in Children and Adolescents: Growth Hormone Deficiency, Idiopathic Short Stature, and Primary Insulin-Like Growth Factor-I Deficiency. Horm. Res. Paediatr..

[34] Rogol A.D., Roemmich J.N., Clark P.A. (2002). Growth at puberty. J. Adolesc. Health.

[35] Fury M.S., Paschos N.K., Fabricant P.D., Anderson C.N., Busch M.T., Chambers H.G., Christino M.A., Cordasco F.A., Edmonds E.W., Ganley T.J. (2022). Assessment of Skeletal Maturity and Postoperative Growth Disturbance After Anterior Cruciate Ligament Reconstruction in Skeletally Immature Patients: A Systematic Review. Am. J. Sports Med..

[36] Marshall W.A., Tanner J.M. (1970). Variations in the pattern of pubertal changes in boys. Arch. Dis. Child..

[37] Huang S., Su Z., Liu S., Chen J., Su Q., Su H., Shang Y., Jiao Y. (2023). Combined assisted bone age assessment and adult height prediction methods in Chinese girls with early puberty: Analysis of three artificial intelligence systems. Pediatr. Radiol..

[38] Park K.W., Kim J.H., Sung S., Lee M.Y., Song H.R. (2014). Assessment of skeletal age in multiple epiphyseal dysplasia. J. Pediatr. Orthop..

[39] Greulich W.W., Pyle S.I. (1959). Radiographic Atlas of Skeletal Development of the Hand and Wrist.

[40] Sanders J.O., Khoury J.G., Kishan S., Browne R.H., Mooney J.F., Arnold K.D., McConnell S.J., Bauman J.A., Finegold D.N. (2008). Predicting scoliosis progression from skeletal maturity: A simplified classification during adolescence. J. Bone Jt. Surg. Am..

[41] Bilgili Y., Hizel S., Kara S.A., Sanli C., Erdal H.H., Altinok D. (2003). Accuracy of skeletal age assessment in children from birth to 6 years of age with the ultrasonographic version of the Greulich-Pyle atlas. J. Ultrasound Med..

[42] Gilli G. (1996). The assessment of skeletal maturation. Horm. Res..

[43] Pierce T.P., Issa K., Festa A., Scillia A.J., McInerney V.K. (2017). Pediatric Anterior Cruciate Ligament Reconstruction: A Systematic Review of Transphyseal Versus Physeal-Sparing Techniques. Am. J. Sports Med..

[44] Faunø P.Z., Bøge Steinmeier Larsen J., Nielsen M.M., Hellfritzsch M., Nielsen T.G., Lind M. (2023). The Risk of Growth Disturbance Is Low After Pediatric Anterior Cruciate Ligament Reconstruction with a Femoral Growth Plate Sparing Technique. Arthrosc. Sports Med. Rehabil..

[45] Kaeding C.C., Flanigan D., Donaldson C. (2010). Surgical techniques and outcomes after anterior cruciate ligament reconstruction in preadolescent patients. Arthroscopy.

[46] van Rijn R.R., Grootfaam D.S., Lequin M.H., Boot A.M., van Beek R.D., Hop W.C., van Kuijk C. (2004). Digital radiogrammetry of the hand in a pediatric and adolescent Dutch Caucasian population: Normative data and measurements in children with inflammatory bowel disease and juvenile chronic arthritis. Calcif. Tissue Int..

[47] Cheung J.P., Cheung P.W., Samartzis D., Cheung K.M., Luk K.D. (2016). The use of the distal radius and ulna classification for the prediction of growth: Peak growth spurt and growth cessation. Bone Jt. J..

[48] Sitoula P., Verma K., Holmes L., Gabos P.G., Sanders J.O., Yorgova P., Neiss G., Rogers K., Shah S.A. (2015). Prediction of Curve Progression in Idiopathic Scoliosis: Validation of the Sanders Skeletal Maturity Staging System. Spine.

[49] Canavese F., Charles Y.P., Dimeglio A. (2008). Skeletal age assessment from elbow radiographs. Review of the literature. Chir. Organi Mov..

[50] Lequin M.H., Hop W.C., van Rijn R.R., Bukkems M.C., Verhaak L.L., Robben S.G., Van Kuijk C. (2001). Comparison between quantitative calcaneal and tibial ultrasound in a Dutch Caucasian pediatric and adolescent population. J. Clin. Densitom..

[51] Pedrotti L., Bertani B., Tuvo G., Barone F., Crivellari I., Lucanto S., Redento M. (2010). Evaluation of bone density in infancy and adolescence. Review of medical literature and personal experience. Clin. Cases Miner. Bone Metab..

[52] Shea K.G., Styhl A.C., Jacobs J.C., Ganley T.J., Milewski M.D., Cannamela P.C., Anderson A.F., Polousky J.D. (2016). The Relationship of the Femoral Physis and the Medial Patellofemoral Ligament in Children: A Cadaveric Study. Am. J. Sports Med..

[53] Knapik D.M., Duong M.M., Liu R.W. (2019). Evaluation of Skeletal Maturity Using the Distal Femoral Physeal Central Peak Is Not Significantly Affected by Radiographic Projection. J. Pediatr. Orthop..

[54] Grassi A., Rossi C., Altovino E., Ambrosini L., Adravanti F.M., Assaf A., Borque K., Zaffagnini S. (2025). Differences between bone age and chronological age in patients with open physes and anterior cruciate ligament injury using a Magnetic Resonance Imaging bone age assessment tool of the knee. J. Exp. Orthop..

[55] Tang H.C., Dienst M. (2020). Surgical Outcomes in the Treatment of Concomitant Mild Acetabular Dysplasia and Femoroacetabular Impingement: A Systematic Review. Arthroscopy.

[56] Milewski M.D., Beck N.A., Lawrence J.T., Ganley T.J. (2011). Anterior cruciate ligament reconstruction in the young athlete: A treatment algorithm for the skeletally immature. Clin. Sports Med..

[57] Perkins C.A., Willimon S.C. (2020). Pediatric Anterior Cruciate Ligament Reconstruction. Orthop. Clin. N. Am..

[58] Zbojniewicz A.M., Meyers A.B., Wall E.J. (2016). Post-operative imaging of anterior cruciate ligament reconstruction techniques across the spectrum of skeletal maturity. Skelet. Radiol..

[59] Willson R.G., Kostyun R.O., Milewski M.D., Nissen C.W. (2018). Anterior Cruciate Ligament Reconstruction in Skeletally Immature Patients: Early Results Using a Hybrid Physeal-Sparing Technique. Orthop. J. Sports Med..

[60] Seilern Und Aspang J., Serrano-Dennis J., Hammond K.E., Slone H.S., Garry J.G., Petit C., Myer G.D., Seguin D., Xerogeanes J.W. (2025). Midterm Outcomes of Hybrid Transepiphyseal ACL Reconstruction with Soft Tissue Quadriceps Tendon Autograft in Skeletally Immature Athletes. Orthop. J. Sports Med..

[61] Pascual-Leone N., Gross P.W., Meza B.C., Fabricant P.D. (2022). Techniques in Pediatric Anterior Cruciate Ligament Reconstruction. Arthroscopy.

[62] Chambers C.C., Monroe E.J., Allen C.R., Pandya N.K. (2019). Partial Transphyseal Anterior Cruciate Ligament Reconstruction: Clinical, Functional, and Radiographic Outcomes. Am. J. Sports Med..

[63] Calvo R., Figueroa D., Gili F., Vaisman A., Mocoçain P., Espinosa M., León A., Arellano S. (2015). Transphyseal anterior cruciate ligament reconstruction in patients with open physes: 10-year follow-up study. Am. J. Sports Med..

[64] Anderson A.F. (2003). Transepiphyseal replacement of the anterior cruciate ligament in skeletally immature patients. A preliminary report. J. Bone Jt. Surg. Am..

[65] Collins M.J., Arns T.A., Leroux T., Black A., Mascarenhas R., Bach B.R., Forsythe B. (2016). Growth Abnormalities Following Anterior Cruciate Ligament Reconstruction in the Skeletally Immature Patient: A Systematic Review. Arthroscopy.

[66] Anderson C.N., Anderson A.F. (2017). Management of the Anterior Cruciate Ligament-Injured Knee in the Skeletally Immature Athlete. Clin. Sports Med..

[67] Shaw L., Finch C.F. (2017). Trends in Pediatric and Adolescent Anterior Cruciate Ligament Injuries in Victoria, Australia 2005–2015. Int. J. Environ. Res. Public Health.

[68] Willimon S.C., Jones C.R., Herzog M.M., May K.H., Leake M.J., Busch M.T. (2015). Micheli Anterior Cruciate Ligament Reconstruction in Skeletally Immature Youths: A Retrospective Case Series with a Mean 3-Year Follow-Up. Am. J. Sports Med..

[69] Moksnes H., Engebretsen L., Risberg M.A. (2012). Management of anterior cruciate ligament injuries in skeletally immature individuals. J. Orthop. Sports Phys. Ther..

[70] Chambers C.C., Zhang A.L. (2019). Outcomes for Surgical Treatment of Femoroacetabular Impingement in Adults. Curr. Rev. Musculoskelet. Med..

[71] Seil R., Theisen D., Moksnes H., Engebretsen L. (2018). ESSKA partners and the IOC join forces to improve children ACL treatment. Knee Surg. Sports Traumatol. Arthrosc..

[72] Ardern C.L., Ekås G., Grindem H., Moksnes H., Anderson A., Chotel F., Cohen M., Forssblad M., Ganley T.J., Feller J.A. (2018). 2018 International Olympic Committee consensus statement on prevention, diagnosis and management of paediatric anterior cruciate ligament (ACL) injuries. Knee Surg. Sports Traumatol. Arthrosc..

[73] Sugimoto D., Del Bel M., Butler L. (2022). Barriers and Facilitators of Research in Pediatric Sports Medicine Practitioners: A Survey of the PRiSM Society. Int. J. Sports Phys. Ther..

[74] Breen A.B., Steen H., Pripp A., Gunderson R., Sandberg Mentzoni H.K., Merckoll E., Zaidi W., Lambert M., Hvid I., Horn J. (2022). A comparison of 3 different methods for assessment of skeletal age when treating leg-length discrepancies: An inter- and intra-observer study. Acta Orthop..

[75] Brix N., Ernst A., Lauridsen L.L.B., Parner E., Støvring H., Olsen J., Henriksen T.B., Ramlau-Hansen C.H. (2019). Timing of puberty in boys and girls: A population-based study. Paediatr. Perinat. Epidemiol..

[76] Moksnes H., Grindem H., Risberg M.A. (2022). Managements of ACL injuries in skeletally immature athletes. Br. J. Sports Med..

[77] Nguyen J.C., Guariento A., Nicholson A., Nguyen M.K., Gendler L., Ho-Fung V., Zhu X., Talwar D., Darge K., Flynn J.M. (2021). Hand Bone Age Radiography: Comparison Between Slot-scanning and Conventional Techniques. J. Pediatr. Orthop..

[78] Dekker T.J., Rush J.K., Schmitz M.R. (2018). What’s New in Pediatric and Adolescent Anterior Cruciate Ligament Injuries?. J. Pediatr. Orthop..

[79] Kocher M.S., Heyworth B.E., Fabricant P.D., Tepolt F.A., Micheli L.J. (2018). Outcomes of Physeal-Sparing ACL Reconstruction with Iliotibial Band Autograft in Skeletally Immature Prepubescent Children. J. Bone Jt. Surg. Am..

[80] Webster K.E., Feller J.A., Leigh W.B., Richmond A.K. (2014). Younger patients are at increased risk for graft rupture and contralateral injury after anterior cruciate ligament reconstruction. Am. J. Sports Med..

[81] Shaw L., Finch C.F., Salmon L.J. (2021). Early surgical reconstruction versus rehabilitation for ACL injuries in young athletes. Sports Med..

[82] Mouton C., Moksnes H., Janssen R., Fink C., Zaffagnini S., Monllau J.C., Ekås G., Engebretsen L., Seil R. (2021). Preliminary experience of an international orthopaedic registry: The ESSKA Paediatric Anterior Cruciate Ligament Initiative (PAMI) registry. J. Exp. Orthop..

[83] Moksnes H., Engebretsen L., Seil R. (2016). The ESSKA paediatric anterior cruciate ligament monitoring initiative. Knee Surg. Sports Traumatol. Arthrosc..

